# Massive pelvic recurrence of uterine leiomyomatosis with intracaval-intracardiac extension: video case report and literature review

**DOI:** 10.1186/s12893-017-0306-y

**Published:** 2017-11-29

**Authors:** Lidia Castagneto Gissey, Germano Mariano, Layla Musleh, Pasquale Lepiane, Marco Colasanti, Roberto L. Meniconi, Federico Ranocchi, Francesco Musumeci, Mario Antonini, Giuseppe M. Ettorre

**Affiliations:** 1 Division of General Surgery and Liver Transplantation, San Camillo-Forlanini Hospital POIT - L. Spallanzani INMI, Rome, Italy; 2 Department of Surgical Sciences, Sapienza University of Rome, Viale del Policlinico 155, 00161 Rome, Italy; 3 Department of Cardiac Surgery, San Camillo-Forlanini Hospital, Rome, Italy; 4 Department of Anesthesia and Critical Care, INMI L. Spallanzani I.R.C.C.S, Rome, Italy

**Keywords:** Intracardiac leiomyomatosis, Intravenous leiomyomatosis, Uterine leiomyoma, Hysterectomy, Cardiopulmonary bypass

## Abstract

**Background:**

Uterine leiomyomas represent the gynecological neoplasm with the highest prevalence worldwide. This apparently benign pathological entity may permeate into the venous system causing the so-called intravenous leiomyomatosis of the uterus (IVL). IVL may seldom extend to large caliber veins and reach the right cardiac chambers or pulmonary arteries and cause signs of right sided congestive heart failure and sudden death. Due to its low incidence, however, IVL with intracardiac extension is often misdiagnosed resulting in deferred treatment. No consensus has been obtained regarding the standard surgical approach to be used for this rare condition. We describe the case of a massive pelvic recurrence of uterine leiomyomatosis with intracardiac extension and provide a review of the literature, analyzing management and surgical outcomes.

**Case presentation:**

We present the case of a 46-year-old premenopausal woman presenting with lower-extremity edema, recurrent syncopes and a history of subtotal hysterectomy for multiple uterine fibroids. She was diagnosed with pelvic recurrence of uterine leiomyomatosis and IVL with cardiac involvement. A two-stage surgical excision of the intracardiac-intracaval mass and pelvic leiomyomatosis was performed. The patient had an uneventful recovery and no evidence of recurrence was observed on follow-up.

**Conclusions:**

By virtue of the rarity of the present pathology, awareness is widely scarce and diagnosis is often delayed. Early recognition is difficult due to initial aspecific and subtle clinical manifestations. Nevertheless, suspicion should be held high in premenopausal women with known history of uterine leiomyomata, presenting with cardiovascular symptoms and evidence of a free-floating mass within the right cardiac chambers. In-depth imaging is crucial for defining its anatomical origin and relations. Prompt surgical treatment with radical excision of pelvic and intravenous leiomyomatosis guarantees favorable outcomes and excellent prognosis with low rates of recurrence, whereas delayed diagnosis and treatment exposes to increased risk of congestive heart failure and sudden death.

**Electronic supplementary material:**

The online version of this article (10.1186/s12893-017-0306-y) contains supplementary material, which is available to authorized users.

## Background

Representing the gynecological neoplasm with the highest prevalence worldwide, uterine leiomyomas affect approximately 70–80% of women in premenopausal age [1, 2]. Broadly known under the nomenclature of myomas or fibroids, leiomyomas globally account for the leading indication for hysterectomy [3]. The exact incidence of such tumors, however, has yet to be determined in consideration of the large number of asymptomatic, thus undiagnosed cases.

Uterine fibroid tumors derive from a benign monoclonal proliferation of smooth muscle cells of the uterine myometrium. An exact etiopathogenesis, nevertheless, has not been fully elucidated. A variety of risk factors, ranging from genetic predisposition to ethnicity, hormonal factors, nulliparity, and obesity have been recognized as key players in the development of uterine leiomyomata. Additionally, its estrogenic dependence has been well characterized and confirmed by its increased occurrence during reproductive age [4].

Specific mention should be owed, as a separate pathological entity, to the so called intravenous leiomyomatosis of the uterus (IVL). This infrequent condition, initially delineated by Birch-Hirschfeld in 1896 [5], is characterized by the abnormal growth of a benign smooth muscle cell tumor arising from the uterine venous system, which may subsequently propagate further to greater caliber veins and occasionally reach the cardiac chambers. Durck and Hörmann reported of two distinct but analogous cases of leiomyomatous tissue extending from the uterus to the right atrium by means of the inferior vena cava (IVC), both of which ended in fatal outcomes and were noted at autopsy [6]. Although histopathologically benign, this type of neoplasm is endowed with a highly aggressive comportment, in view of its tendency to invade the venous system, extending to large caliber vessels and finally to the heart and pulmonary arteries, leading to *exitus* if left untreated.

Mechanisms by which IVL occurs have yet to be defined. Hypotheses have been postulated to tentatively explain its pathogenesis. Knauer suggested that the intravenous masses originated from the proliferation of smooth muscle cells of the vessel’s media, in consideration of the endothelial coverage of the masses at microscopic examination [7]. In contrast, Sitzenfrey proposed that myomatous tissue could directly permeate into the lumen of contiguous blood vessels [8]. Etiopathogenesis, however, remains still at present an area of controversy.

Herein, we describe the case of a woman in premenopausal age, presenting with signs and symptoms of right-sided congestive heart failure and a previous history of uterine fibroids, providing a review of the literature with regards to intravenous leiomyomatosis of the uterus with cardiac involvement.

### Literature review

A review of the English literature of all fully described cases of IVL with intracardiac involvement was performed, using the following key words: *intravenous leiomyomatosis, intracardiac leiomyomatosis, diagnosis, management, surgical approach.* A total of 198 articles were found and reviewed by two independent researchers; 102 articles of intracardiac leiomyomatosis of uterine origin were identified as adequately describing in depth the clinical cases.

## Case presentation

A 46-year-old Caucasian woman, with a body mass index (BMI) of 20.7 kg/m^2^ and absence of risk factors for cardiovascular disease, nulligravida, nulliparous, was admitted to the Emergency Department of the San Camillo Hospital in Rome (Italy), presenting with abdominal swelling associated with exertional dyspnea and recurrent syncopes. On physical examination the patient showed lower-extremity edema, jugular venous distension and grade III/VI tricuspidal holosystolic murmur.

Her past medical history was significant for uterine leiomyomata for which she underwent multiple myomectomies. Because of a progressive, aberrant growth of the myomatous uterus, the patient subsequently received a subtotal hysterectomy with preservation of the cervix and adnexae, approximately 1 year prior to the present scenario. On histopathological examination, ordinary uterine leiomyomas were found, with the peculiar aspect of microscopic, thread-like masses into the lumina of small caliber venous structures confined to the resected uterus.

**Fig. 1:**
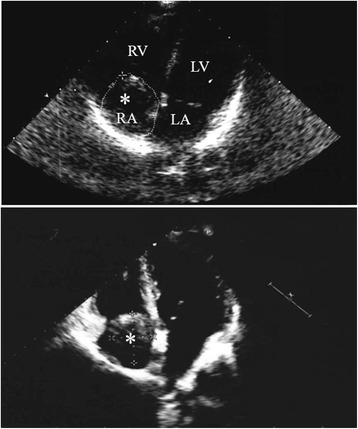
Transthoracic two-dimensional echocardiography revealing a fluctuating solid mass (*asterisk*) within the right atrium (*RA*) (5 × 3.5 cm), ascending from the inferior vena cava Right ventricle (*RV*); *left* ventricle (*RL*); *left* atrium (*LA*)

**Fig. 2:**
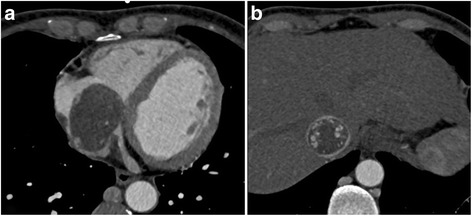
Cardiac CT scan showing a hypodense mass occupying the *right* atrium (**a**) and the inferior vena cava in toto (**b**)

On admission the patient underwent a two-dimensional Doppler transthoracic echocardiography followed by a cardiac computed tomography which showed the presence of a 5 × 3.5 cm fluctuating solid, thrombus-like mass inside the right atrium and ascending from the IVC (Figs. 1 and 2).

**Fig. 3:**
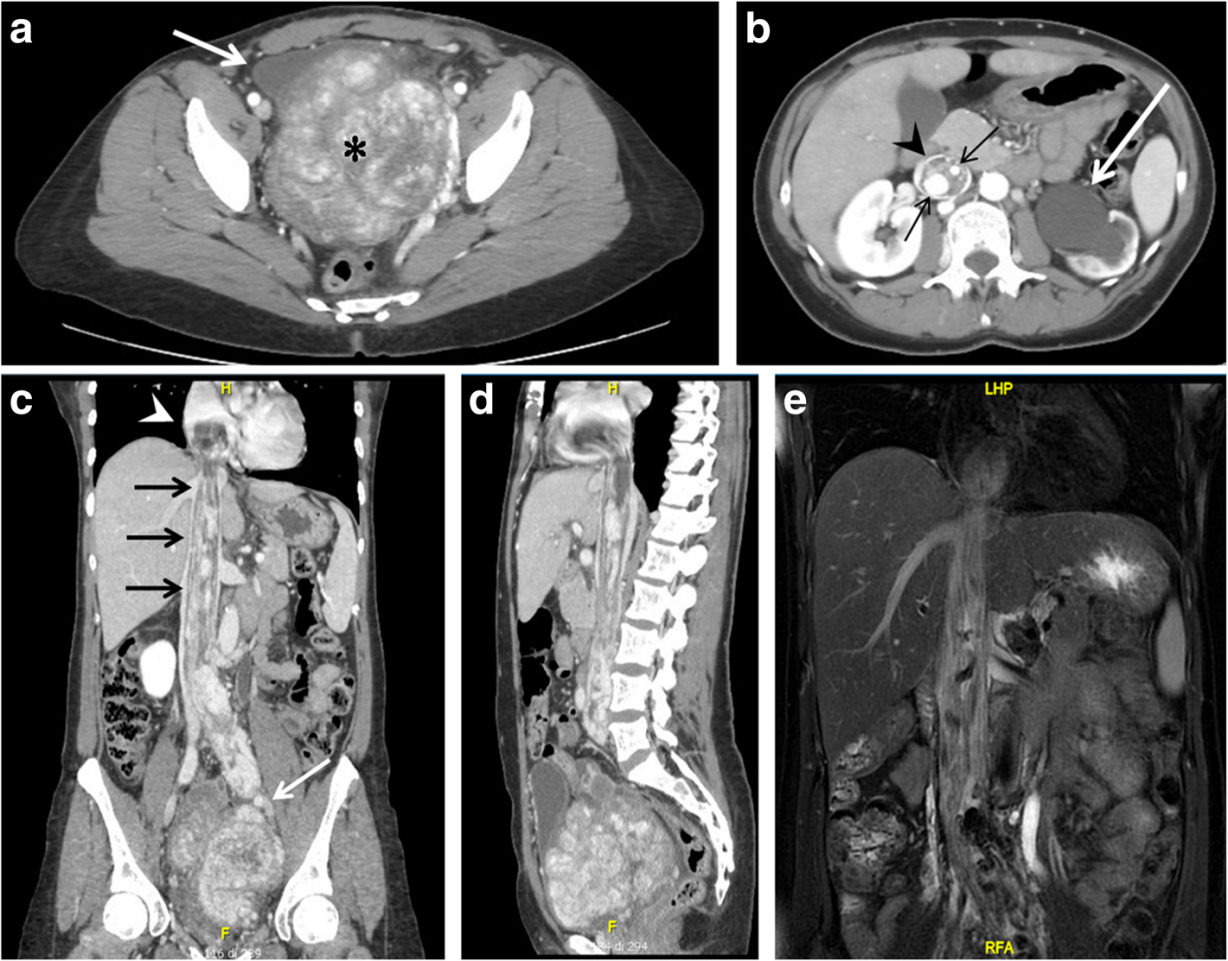
Chest – abdomen – pelvis CT scan (**a-d**) and MR (**e**). **a.** Axial projection showing a voluminous pelvic mass of 15 × 22 × 9 cm (*asterisk*), with significant contrast enhancement due to hypervascularization, causing *right* antero-lateral dislocation of the bladder (*arrow*). **b**. The mass also determines left hydro-ureteronephrosis (*white arrow*). Notice the presence of contrast-enhanced large vessels (*black arrows*) within the IVC (*arrowhead*). **c-d**. Coronal and sagittal CT projections showing the pelvic tumor directly extending into the left iliac vein (*white arrow*), IVC (*black arrows*) and occupying the right atrium (*arrowhead*). **e**. Coronal MR projection showing the caval and cardiac extension of the tumor

Successively, a chest-abdomen-pelvis Computed Tomography (CT) and Magnetic Resonance (MR) revealed a voluminous (15 × 22 × 9 cm) pelvic mass that caused compression of the bladder and left ureter, determining severe left hydroureteronephrosis (Fig. 3a-b). Moreover, inside the lumen of the left iliac vein and inferior vena cava, finally extending into the right atrium, a solid mass, provided with large vessels within, was noted (Fig. 3b-e).

### Therapeutic management

Low molecular weight heparin (12,000 IU per day) was commenced and diuretic therapy set out. Improvement of lower-extremity edema and of dyspnea was observed.

**Fig. 4:**
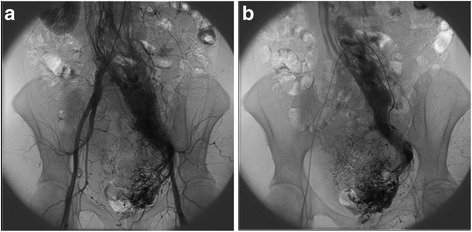
Arteriogram showing (**a**) bilateral hypogastric artery dilation, with (**b**) marked left-sided hypogastric artery prominence and tumor hypervascularization

Bilateral uterine artery embolization was performed preoperatively to prevent mass growth and reduce the risk of copious intraoperative bleeding. Arteriography of the hypogastric arteries showed bilateral dilation with marked left-sided prominence and an altered, protuberant vascularity of the neoformed pelvic mass (Fig. 4). Additionally, cystoscopy with retrograde bilateral double J stenting was carried out to treat the left hydroureteronephrosis caused by *ab estrinseco* compression of the left ureter. Furthermore, bilateral stenting was also used for prevention of iatrogenic injury, granting intraoperative guidance to ureteral identification, in consideration of the high complexity of the pelvic pathology.

In view of the extension of both the pelvic and intravascular masses, the patient was scheduled for a two-stage surgical procedure.

### Surgical procedure

**Fig. 5:**
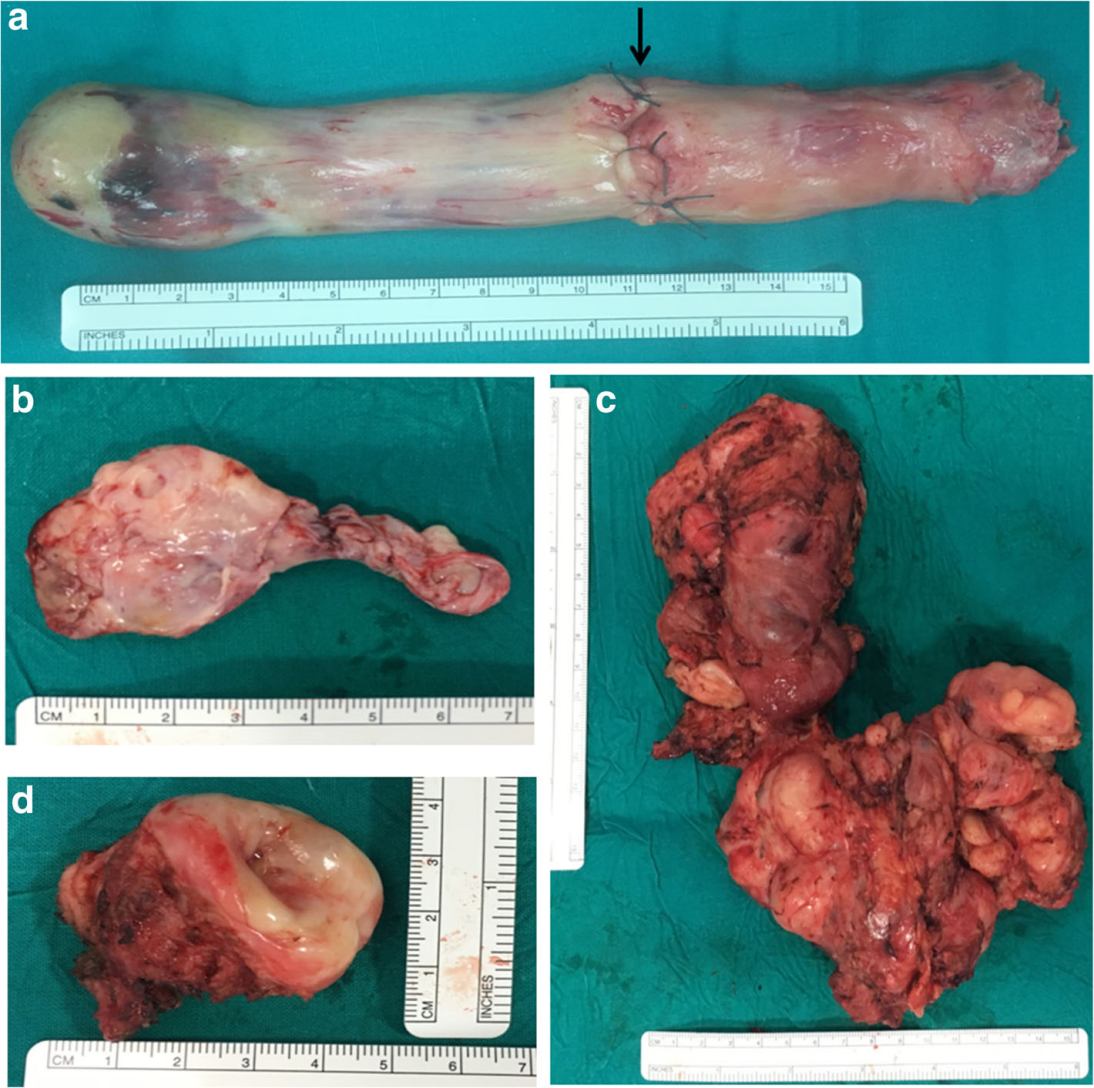
Gross specimen. **a** Intracardiac-intracaval mass measuring 16 × 4 × 3 cm with transection line at infrarenal level (*arrow*) and lower transection at supra-iliac level. **b** Iliocaval confluence and *left* iliac vein mass of 8 cm. **c.** Pelvic leiomyomatous mass measuring 15 × 22 × 9 cm. **d.** Cervix (6 × 3.5 × 2 cm)

The first stage of the procedure involved sterno-laparotomy, cardiopulmonary bypass (CPB) with cannulation of the ascending aorta, superior vena cava and right atrial appendage, followed by hypothermia (26 °C), circulatory arrest and right atriotomy. Concurrently, a cavotomy of the IVC at infrarenal level was performed in order to divide the intravenous leiomyoma and allow for a safe extraction through the atriotomy. Closure of the atrial opening was then performed and gradual, uncomplicated weaning from CPB was obtained after patient rewarming. Division was made necessary due to extensive adhesions of the intravenous mass to the inner caval wall. A second cavotomy, superiorly to the iliocaval confluence allowed for removal of the residual intracaval leiomyoma (Fig. 5a).

Second-stage surgery was performed 7 days later and consisted of a relaparotomy, longitudinal cavotomy superiorly to the iliac confluence and extraction of the intravenous leiomyoma occupying the iliocaval bifurcation extending to the left iliac vein (Fig. 5b). ‘En bloc’ resection of the voluminous pelvic leiomyomatous mass with adnexae and cervix was then performed (Fig. 5c and d). Ligation and excision of the left hypogastric vein was also executed in order to radically remove all myomatous intravenous tissue, thus preventing recurrence.

Histopathological examination was consistent with pelvic recurrence of uterine leiomyomatosis with IVL. The intravenous masses were composed of elongated cell bundles with areas of hyalinization and a large amount of collagenous fibers amidst; such cells expressed smooth muscle cell markers on immunohistochemical analysis (i.e. smooth muscle actin, desmin), confirming their leiomyomatous origin.

The patient had an uneventful recovery, length of stay in intensive care unit was 3 days and the patient was then discharged from our department on postoperative day 9.

**Fig. 6:**
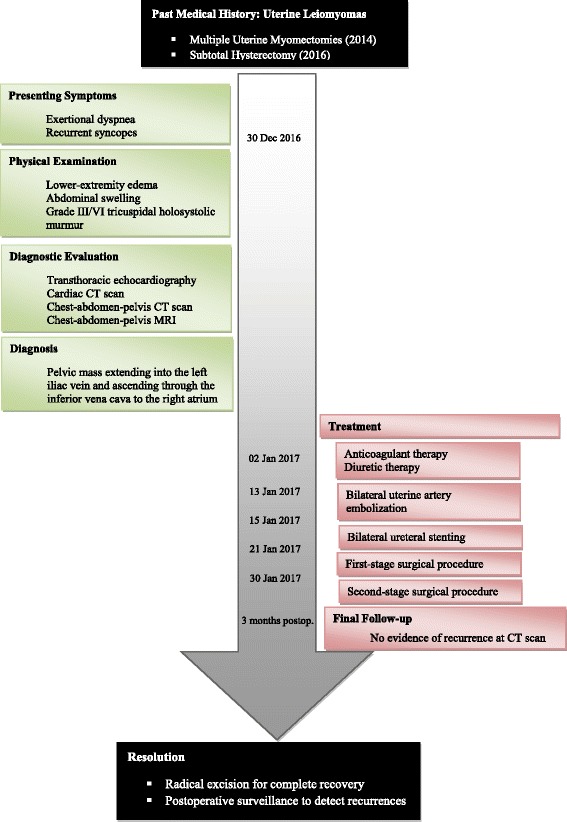
Clinical case timeline

At 3 month follow-up the patient did not show evidence of disease recurrence. Nonetheless, long-term surgical follow-up and surveillance was advised (Fig. 6).

An additional movie file shows the two-stage surgical procedure in detail (Additional file 1).

## Results of literature review

Mean age, race, previous pregnancies, previous uterine surgeries, presenting symptoms, site of venous invasion, cardiac extent and therapeutic management are reported in Table 1.

**Table 1:** Intracardiac leiomyomatosis of uterine origin: summary of reported cases in English literature

Total patients (n)	109
Mean age (years)	47.82 ± 10.00
Race (%)	
Caucasian	31 (28.4)
Asian	56 (51.4)
African	2 (1.8)
Hispanic	4 (3.7)
Not stated	15 (13.8)
Previous pregnancies (%)	
Parous	25 (22.9)
Nulliparous	10 (9.2)
Ongoing	2 (1.8)
Not reported	71 (65.1)
Previous uterine surgery (%)	
Partial hysterectomy	6 (5.5)
Total hysterectomy	21 (19.3)
Hysterectomy and BSO	7 (6.4)
Hysterectomy and USO	7 (6.4)
Myomectomy	9 (8.3)
None	54 (49.5)
Other	5 (4.6)
Presenting Symptoms (%)	
Abnormal uterine bleeding	10 (9.2)
Lower-extremity edema	22 (20.2)
Abdominal distension	4 (3.7)
Abdominal-pelvic pain	10 (9.2)
Chest pain	13 (11.9)
Dyspnea	40 (36.7)
Sudden death	1 (0.9)
Palpitations	11 (10.0)
Asymptomatic	14 (12.8)
Other	5 (4.6)
Site of venous invasion (%)	
Left gonadal/iliac vein	27 (24.8)
Right gonadal/iliac vein	51 (46.8)
Bilateral	12 (11.0)
Unknown	19 (17.4)
Cardiac extent (%)	
Right atrium	64 (58.7)
Right ventricle	33 (30.2)
Pulmonary artery	12 (11.0)
Pulmonary nodules	3 (2.8)
Therapeutic intervention (%)	
Partial resection	14 (12.8)
Complete resection	
One-stage procedure	48 (44.0)
Two-stage procedure	44 (40.3)
Uterine embolization	1 (0.9)
Hormonal therapy	4 (3.7)
None	1 (0.9)
Recurrence rate (%)	
Complete resection	1 (1.1)
Incomplete resection	4 (28.6)

Age is reported as mean ± SD. Data are reported as absolute numbers and percentage in brackets

*USO* Unilateral salpingo-oophorectomy, *BSO* Bilateral salpingo-oophorectomy

## Discussion

IVL is an uncommon, histologically benign neoplasm usually confined to the venous system of the uterus and is most often incidentally diagnosed during histopathological analysis of specimen deriving from uterine surgery (i.e. myomectomy, hysterectomy, etc). However, it may seldom grow into large-caliber systemic veins and ultimately reach the heart. At this level it can lead to right-sided congestive heart failure and eventually death.

Intra-cardiac leiomyomatosis is a serious, advanced condition that, if promptly diagnosed and surgically excised, permits a rapid recovery of right cardiac function and may have a positive prognosis. The analysis of the literature (Table 1) shows that the age of IVL onset is approximately 48 years and that Asian (51.4%) followed by Caucasian (28.4%) are the most frequently affected ethnicities. Previous pregnancies are reported in about 23% of cases and previous total hysterectomy in 19%. The most frequent presenting symptoms are those related to right cardiac failure, with dyspnea in about 37%, lower extremity edema in 20%, chest pain in 12% and palpitation in 10% of cases; however, 13% of the patients are asymptomatic. Right atrium is invaded in 59% whereas right ventricle in 30% of cases. Complete resection is equally performed by one or two stage procedures.

IVL mainly involves premenoupasal women - although age is highly variable and ranges from 21 to 80 years old - of Caucasian race, usually reporting a past history of uterine fibroids. The clinical picture of patients presenting with intra-cardiac dissemination of IVL usually includes dyspnea, thoracic pain, syncope and lower-extremity edema in addition to menometrorrhagia, abdominal-pelvic discomfort or pain and abdominal swelling, directly related to the volume of the pelvic mass [9].

We describe the case of a 46-year-old woman affected by IVL with a voluminous intracaval-intracardiac mass almost completely occupying the right atrium and causing signs and symptoms of right cardiac failure. She was admitted to the Department of Emergency Medicine of our hospital with the diagnosis of a free-floating atrial mass, initially mistaken for a thrombus. She was then referred to our Division of General Surgery where she was accurately studied, IVL with cardiac involvement was recognized and surgery was scheduled.

The radical resection of the pelvic mass in association with the extraction of the intra-iliac, intra-caval and intra-cardiac extension of the tumor proved to be a remedial action for the cardiovascular symptoms with complete recovery of the patient.

In advanced conditions with involvement of cardiac chambers or pulmonary arteries, a holistic, multidisciplinary approach combining general and cardiac surgery is imperative in order to guarantee a safe and complete removal of the leiomyomatous mass.

Radical surgical excision is recommended and should always include total abdominal hysterectomy and bilateral salpingo-oophorectomy or in case of previous uterine surgery, a totalization of the hysterectomy and removal of any pelvic recurrence with total resection of the IVL extensions. Bilateral salpingo-oophorectomy is of cardinal importance in light of the estrogen-sensitivity of the tumor [10].

While complete resection is rarely associated with tumor recurrence [11], an incomplete excision increases the risk of neoplasm reappearance in 1/3 of cases [12]. Considering recurrence rates and rapid growth of IVL, postoperative surveillance is essential in order to promptly detect cases of recurrence and allow for immediate therapeutic action.

A surgical resection in one or two stages is equally effective with a similar incidence of postoperative complications [13–17]. Thus, preference towards the choice of a single or double-staged intervention should be tailored on each distinct patient and based on the general health status in addition to the extent of pelvic and intravenous pathology. In the present case, a single-stage operation, in consideration of a previous surgical history positive for multiple pelvic operations, implying extensive pelvic adhesions, would have been at higher risk of local bleeding due to the anticoagulation state needed during cardio-pulmonary bypass. We therefore opted for a staged approach. Conversely, we have previously reported the case of a female patient presenting with an analogous condition where a one-stage procedure was adopted, in view of the inferior complexity of the pelvic component [18]. The patient has completed now a 36 month follow-up and has shown no evidence of disease.

Differential diagnosis is of fundamental importance and should be made in the first instance with atrial myxoma, a primary heart tumor, although typically localized in the left atrium. Moreover, differential diagnosis should include a thrombus-in-transit and several malignant conditions with direct IVC tumor invasion such as renal cell carcinoma, hepatocellular carcinoma, adrenocortical carcinoma and lymphoma.

By virtue of the rarity of the present pathology, awareness is widely scarce and diagnosis is often deferred. This leads to IVL recognition in advanced stages when cardiac involvement and cardiovascular repercussions have already established. Early diagnosis is difficult due to initial aspecific and subtle clinical manifestations. However, suspicion should be held high in premenopausal female patients with known history of leiomyomata of the uterus, cardiovascular symptomatology and evidence of a mass within the right cardiac chambers and should be further studied through CT scan or MR so as to define its anatomical origin and relations.

## Conclusion

In conclusion, in presence of signs and symptoms of right-sided heart failure and evidence of a free-floating right atrial mass at imaging, in a premenopausal woman with a history of uterine leiomyomas, IVL should always be suspected and considered for differential diagnosis. Prompt surgical treatment with radical excision of pelvic and intravenous leiomyomatosis guarantees favorable outcomes and excellent prognosis with low rates of recurrence, whereas delayed diagnosis and treatment exposes to increased risk of congestive heart failure and sudden death.

## Additional file

Additional file 1: Video link: https://drive.google.com/file/d/0B6Pduws2F4kTWUZEdUE4ZDBwRUU/view?usp=sharing. File format: avi. Title: Massive Pelvic Recurrence of Uterine Leiomyomatosis with Intracaval-Intracardiac. Extension: Video Case Report and Literature Review. Description: Video case report illustrating in detail the two-stage surgical procedure of intracardiacintracaval IVL removal followed by IVL extraction from the iliocaval confluence and left hypogastric vein in addition to radical excision of the pelvic recurrent mass. (Stage 1: operative time 372’; CPB 190’; intraoperative blood loss 550 ml; PRBCs transfusions 5 Units. Stage 2: operative time 302’; intraoperative blood loss 1500 ml; PRBCs transfusions 4 Units). (ZIP 52077 kb)

## Abbreviations

BMI: Body mass index
BSO: Bilateral salpingo-oophorectomy
CPB: Cardiopulmonary bypass
CT: Computed tomography
IVC: Inferior vena cava
IVL: Intravascular leiomyomatosis
MR: Magnetic resonance
USO: Unilateral salpingo-oophorectomy
